# Partial remission of acute myeloid leukemia complicating multiple myeloma following COAP chemotherapy: A case report

**DOI:** 10.3892/ol.2015.2867

**Published:** 2015-01-12

**Authors:** MAN SHEN, WAN-JUN SUN, ZHONG-XIA HUANG, JIA-JIA ZHANG, NA AN, XIN LI

**Affiliations:** 1 Department of Hematology and Oncology, Beijing Chaoyang Hospital, Capital Medical University, Beijing 100043, P.R. China; 2 Department of Hematology, The Second Artillery General Hospital, Beijing 100088, P.R. China

**Keywords:** acute myeloid leukemia, multiple myeloma, partial remission, COAP

## Abstract

A 77-year-old male was admitted to hospital after complaining of fever and a cough for three days. A diagnosis of multiple myeloma was confirmed following M protein identification and a bone marrow biopsy. The patient received chemotherapy regimens of bortezomib plus dexamethasone, cyclophosphamide, thalidomide and dexamethasone, and thalidomide and dexamethasone, and was prescribed thalidomide (100 mg/d) to be taken orally for maintenance therapy. After a further two years the patient was subsequently diagnosed with acute myeloid leukemia. Chemotherapy regimens of cytarabine, aclacinomycin and daunorubicin, homoharringtonine and etoposide, and mitoxantrone and cytarabine resulted in no remission. Partial remission was obtained with a course of ifosfamide, vindesine, cytarabine and prednisone chemotherapy. This therapy may be an alternative treatment for secondary leukemia, particularly in elderly patients.

## Introduction

Multiple myeloma (MM) survival rates have improved significantly over the last 10 years (1–3). For example, in subsequent 10-year calendar periods, the median overall survival increased from 24.3 to 56.3 months (P=0.036) in patients ≤65 years old. With such improvements, a relatively new clinical challenge that has emerged is the risk of second malignancies. The association of acute leukemia [most frequently acute myeloid leukemia (AML)] and MM has often been reported, not only as a complication of chemotherapy, but also occurring in the absence of chemotherapy or simultaneously at the time of diagnosis (4). Prior studies have found that various therapies have made a contribution to secondary malignancies following multiple myeloma (5). The therapies included oral alkylating therapy (6–13), myeloablative therapy (used in conjunction with ASCT) (10–12), radiotherapy (14–16) and lenalidomide (17–19). There are several ongoing studies are attempting to understand the underlying mechanisms. Several studies have shown that the rate of hematological malignancy after multiple myeloma was 0.5%–12.2%. Reece and Goswami reported that the rate of MDS/AML after diagnosis of multiple myeloma of initiation of lenalidomide was 2.6%. Treatment of patients with two hematological cancers is difficult. The current study reports a case of MM complicated by AML. The patient received classic chemotherapy regimens including cytarabine, aclacinomycin, daunorubicin (CAG), mitoxantrone and cytarabine (NA) and homoharringtonine and etoposide (HE), however, did not respond to the treatment. A course of COAP chemotherapy was subsequently administered, which was selected as it is frequently used to treat AML and has been well-tolerated by patients, with few side effects. Guthrie reported that fifteen AML patients had received chemotherapy with a combination of high-dose, continuous-infusion COAP (20). The results showed that the rate of complete remission was 47% and the rate of partial remission was 40%. The main toxicity was primarily myelosuppression and there were no toxicities such as hemorrhagic cystitis or central nervous system, hepatic or pulmonary toxicity. Written informed consent was obtained from the patient’s family.

## Case report

A 77-year-old male was admitted to the Department of Hematology, Peking Union Medical College Hospital (Beijing, China) on June 21st, 2011, after presenting with fever and a cough persisting for three days. Following hospitalization, laboratory tests revealed a white blood cell count of 3,900/mm^3^ (normal range, 4,000–10,000/mm^3^), a hemoglobin level of 128 g/l (normal range, 131–172 g/l), a platelet count of 54,000/mm^3^ (normal range, 100,000–300,000/mm^3^), and a serum creatinine level of 133 μmol/l (normal range, 53–115 μmol/l). Further analysis revealed a blood microglobulin level of 4.53 mg/l (normal range, 1.0–3.0 mg/l), a blood sedimentation rate of 21 mm/h (normal range, 0–20 mm/h), and M protein levels of 6.78 g/l for IgG (normal range, 7.51–15.6 g/l), 0.22 g/l for IgA (normal range, 0.82–4.53 g/l) and 0.26 g/l for IgM (normal range, 0.46–3.04 g/l). The serum free light chain (κ and λ) levels were measured as 6.52 g/l (κ) and 2.16 g/l (λ), and the total amount of κ measured in the urine was 7.5 g in 24 h. Myeloma cells accounted for 12.5% of bone marrow cytology. A bone marrow biopsy showed elevated numbers of plasma cells (12.5%). Immunohistochemistry revealed that the patient was positive for CD38, CD138, κ and epithelial membrane antigen. CD20, CD3, CD79a, Mum-1 were also positive, but scattered, and the positive rate of Ki-67 was 60%. A bone scan revealed uneven uptake of radioactive tracer in the thoracolumbar region. A diagnosis of multiple myeloma κ type light chain (Durie-Salmon stage II B, International Staging System stage III) was determined. The patient received a chemotherapeutic regimen of bortezomib (1.3 mg/m^2^, days 1, 4, 8 and 11) plus dexamethasone (20 mg, days 1, 4, 8 and 11) (PD) for three cycles, cyclophosphamide (500 mg, days 1–4), thalidomide (100 mg/day, every day) and dexamethasone (20 mg, days 1–4) (CTD) for one cycle, and thalidomide (100 mg/day, every day) and dexamethasone (20 mg, days 1–4) (TD) for one cycle, sequentially, where one cycle comprised 21 days. This was followed by a course of thalidomide (100 mg/d) for maintenance therapy. Blood analysis and tests of hepatorenal function and M protein levels were conducted every three months for two years, yielding normal results.

In April 2013, tests revealed a leukocyte count of 20,830/mm^3^ (normal range, 4,000–10,000/mm^3^) and a platelet count of 27,000/mm^3^ (normal range, 100,000–300,000/mm^3^), while the hemoglobin level was measured at 126 g/l (normal range, 131–172 g/l). Bone marrow examination showed clear signs of hyperplasia, with primitive granulocyte cells increasing from 1 to 74% and visible Auer’s bodies. The rate of positive peroxidase staining was 98%, which differentiated the patient’s diagnosis of AML from ALL. CD117, CD38, CD34, CD13, and HLA-DR were all positive in the immune peripheral blood classification. No FLT3/ITD, NPM1, c-kit/D816V and AML1-ETO mutations were identified in the bone marrow by immunohistochemistry. A diagnosis of AML-M_2_ was determined. On April 29th 2013, after receiving the CAG regimen (cytarabine, 10 mg/m^2^ every 12 h, days 1–14; aclarubicin, 10 mg, days 1–8; G-CSF, 200 μg/m^2^, days 1–14) for one cycle as induction chemotherapy, no remission (NR) was observed in the bone marrow biopsy. The patient subsequently received an additional regimen of HE (homoharringtonine, 3 mg, days 1–7; etoposide, 100 mg, days 1–7). During the entire treatment period, the patient had a respiratory infection and pleural effusion, which ultimately improved following aggressive anti-infection treatment.

In July 2013, the patient began attending the Department of Hematology and Oncology at Beijing Chaoyang Hospital (West Branch, Beijing 100043, China) for treatment. Tests revealed that myeloblasts accounted for 79% (normal range, <5%) of bone marrow cytology, while immature plasma cells accounted for 2% (normal range, <5%). This indicated that the patient now had leukemia, but that the multiple myeloma was well controlled at the same time. M protein identification and immunofixation electrophoresis (IFE) examination results indicated a polyclonal immunoglobulin, while M component was not detected. The patient’s previous medical history included tuberculosis and hemoptysis in 1956, hypertension in 2006 and a coronary computed tomography angiogram in 2010 which revealed 70% coronary stenosis. Coronary angiography and stent therapy was recommended, but the patient refused. In 2006, renal function was observed to be abnormal, with an elevated serum creatinine level of 110 μmol/l (normal range, 53–115 μmol/l). After two years, serum creatinine levels had increased to 140–150 μmol/l, while routine urine test results remained normal. In 2010, the patient was diagnosed with diabetes, cataracts and benign prostatic hyperplasia. Chemotherapy with NA (mitoxantrone, 4 mg, days 1–3; cytarabine, 100 mg, days 1–7) was accepted in July 2013 when bone marrow aspiration cytology results revealed that the high proportion of myeloblasts in the bone marrow (57%) had not yet returned to normal. On October 26th 2013, the patient began a chemotherapy cycle of ifosfamide, vindesine, cytarabine and prednisone (COAP) with ifosfamide, 0.5 g from d1 to d4 combined with vindesine, 4 mg, d1; cytarabine, 100 mg and prednisone, 50 mg every other day from d1 to d7. On November 28th 2013, a re-examination of the patient’s bone marrow indicated partial remission, with primitive cells accounting for 14.7% of all nucleated cells (>50% decrease). During this intermittent chemotherapy, the patient exhibited stable vital signs, with no fever, no cough, no sputum, and no evident abnormalities in routine blood test results. The periodic review of IFE was negative, which revealed that the patient had achieved complete remission of MM. Since November 28th, the patient continued COAP chemotherapy for three cycles. During this chemotherapy cycle, the patient exhibited fever, pancytopenia, agranulocytosis and severe anemia as a result of immunosupression induced by the chemotherapy treatment. A lung CT scan revealed pulmonary consolidation in the right upper lobe and possible lung abscess. This was treated with maxipime (2.0 g, twice a day), meropenem (0.5 g, three times a day) combined with vancomycin (1.0 g, twice a day) and teicoplanin (200 mg, twice a day). Candida albicans, detected in pathological examination of the sputum, was treated with caspofungin antifungal therapy (50 mg, once a day). Although the T spot result was negative, the lesions in the right upper lobe combined with a continuing low-grade fever led to a diagnosis of tuberculosis (TB). Isoniazid (0.3 g, once a day) and rifapentine (0.45 g, once a day) were administered orally to treat the TB. Thereafter, the patient’s fever persisted, peaking once per day. One week later, a further lung CT revealed serious pneumonia, airway obstructive pulmonary consolidation and occupying lesions. The hole-like lesions of the right upper lobe had increased markedly in size, with massive pleural effusion on the right side. Anti-inflammatory treatment was adjusted to sulperazon (3.0 g, twice a day) combined with tigecycline (50 mg, twice a day). The patient’s fever persisted throughout this period, and he subsequently succumbed to respiratory failure.

## Discussion

A number of studies have reported an increased risk of second primary malignancies (SPM) following MM diagnosis, which are proposed to be associated with novel anti-myeloma treatments (17–19). The introduction of agents such as thalidomide (5), bortezomib (21,22) and lenalidomide (17,23,24) has improved MM survival rates over the last decade. However, accurate estimates of incidence and pathogenesis of second malignancies following MM are lacking. Razavi *et al* (25) evaluated the risk of SPM among 36,491 MM cases reported to the surveillance, epidemiology and end results program between 1973 and 2008. The authors calculated overall and site-specific standardized incidence ratio (SIR) and 95% confidence intervals (CI) for 2,012 SPM cases diagnosed within the 35-year follow-up. No significant overall risk of SPM was identified (SIR=0.98; 95% CI=0.94–1.02).

Numerous risk factors are associated with SPM, including MM disease-related factors, such as treatment and tumor microenvironment, in addition to host-related processes, such as genetic and environmental factors (26). Early observations indicated that treatment-related factors, such as from melphalan, may be the main cause of the increased incidence of myelodysplastic syndrome/acute leukemia in MM patients (13). Cyclophosphamide was found to be less leukemogenic than melphalan (27). In addition, maintenance therapy has been evaluated in relation to the risk of second malignancies in three recently reported multicenter randomized phase III trials (IFM 2005-02, CALGB 100104, and MM-015) (17–19). In the IFM 2005-02 and CALGB 100104 trials, 5.5 and 6.5% of lenalidomide-treated patients developed second malignancies, compared with 1 and 2.5% in the respective control groups.

The current study reports one case of a patient who developed AML two years after being diagnosed with MM. This patient had received velcade and ifosfamide treatment for three cycles, and continued to take thalidomide for two years thereafter. The cause of AML in this patient was unclear. Studies have reported that thalidomide may also potentiate solid SPMs (26,28). We therefore considered that thalidomide may be a cause of AML. Three clinical trials had shown that lenalidomide and thalidomide maintenance therapy could lead to a higher incidence of second primary malignancies, which is relevant to patients receiving melphalan (17–19). The patient in the present case had oral thalidomide as maintainance therapy for two years and, during this period, the patient was not adminstered any other drugs that could induce a tumor (28). The patient had taken thalidomide orally for two years prior to the diagnosis of AML and had received chemotherapy (PD, CTD, CAG and HE regimens) for seven cycles, however, complete remission was not achieved. After receiving the COAP chemotherapy regimen, the leukemia cells of the bone marrow decreased by >50% and the disease stabilized. A second cycle of COAP chemotherapy was predicted to produce CR, however, the patient subsequently acquired an obstructive pneumonia infection, which may have been associated with chemotherapy treatment. Although vigorous anti-infection treatments, including various antibiotics, antifungal agents and antituberculosis drugs, were administered, the patient succumbed to respiratory failure.

This study showed that the regimen of CA was an efficacious chemotherapy for MM combined with AML. However, the present study also indicated that the use of immunomodulatory drugs as a chemotherapy treatment may increase the risk of SPM development in older patients. Therefore, further studies are required to investigate the association between oral thalidomide and the development of AML.

## References

[1] Turesson I, Velez R, Kristinsson SY, Landgren O (2010). Patterns of improved survival in patients with multiple myeloma in the twenty-first century: a population-based study. J Clin Oncol.

[2] Kumar SK, Rajkumar SV, Dispenzieri A (2008). Improved survival in multiple myeloma and the impact of novel therapies. Blood.

[3] Kristinsson SY, Landgren O, Dickman PW (2007). Patterns of survival in multiple myeloma: a population-based study of patients diagnosed in Sweden from 1973 to 2003. J Clin Oncol.

[4] Dunkley S, Gibson J, Iland H, Li C, Joshua D (1999). Acute promyelocytic leukaemia complicating multiple myeloma: evidence of different cell lineages. Leuk Lymphoma.

[5] Morgan GJ, Davies FE, Gregory WM (2013). Long-term follow-up of MRC Myeloma IX trial: Survival outcomes with bisphosphonate and thalidomide treatment. Clin Cancer Res.

[6] Bergsagel DE, Bailey AJ, Langley GR (1979). The chemotherapy on plasma-cell myeloma and the incidence of acute leukemia. N Engl J Med.

[7] (2000). Acute leukaemia and other secondary neoplasms in patients treated with conventional chemotherapy for multiple myeloma. Eur J Haematol.

[8] Dong C, Hemminki K (2001). Second primary neoplasms among 53,159 haematolymphoproliferative malignancy patients in Sweden, 1958–1996: a search for common mechanisms. Br J Cancer.

[9] Hasskarl J, Ihorst G, De Pasquale D (2011). Association of multiple myeloma with different neoplasms: systematic analysis in consecutive patients with myeloma. Leuk Lymphoma.

[10] Barlogie B, Tricot G, Haessler J (2008). Cytogenetically defined myelodysplasia after melphalan-based autotransplantation for multiple myeloma linked to poor hematopoietic stem-cell mobilization: the Arkansas experience in more than 3,000 patients treated since 1989. Blood.

[11] Przepiorka D, Buadi F, McClune B (2007). Myelodysplastic syndrome after autologous peripheral blood stem cell transplantation for multiple myeloma. Bone Marrow Transplant.

[12] Govindarajan R, Jagannath S, Flick JT (1996). Preceding standard therapy is the likely cause of MDS after autotransplants for multiple myeloma. Br J Haematol.

[13] Cuzick J, Erskine S, Edelman D, Galton DA (1987). A comparison of the incidence of the myelodysplastic syndrome and acute myeloid leukaemia following melphalan and cyclophosphamide treatment for myelomatosis. A report to the Medical Research Council’s working party on leukaemia in adults. Br J Cancer.

[14] Featherstone C, Delaney G, Jacob S, Barton M (2005). Estimating the optimal utilization rates of radiotherapy for hematologic malignancies from a review of the evidence: part II leukemia and myeloma. Cancer.

[15] Berrington de Gonzalez A, Curtis RE, Kry SF (2011). Proportion of second cancers attributable to radiotherapy treatment in adults: a cohort study in the US SEER cancer registries. Lancet Oncol.

[16] Berrington de Gonzalez A, Curtis RE, Gilbert E (2010). Second solid cancers after radiotherapy for breast cancer in SEER cancer registries. Br J Cancer.

[17] Attal M, Cances-Lauwers V (2011). Maintenance treatment with lenalidomide after transplantation for myeloma: analysis of secondary malignancies within the IFM 2005-02 trial.

[18] McCarthy P, Anderson K (2011). Phase III Intergroup study of lenalidomide versus placebo maintenance therapy following single autologous stem cell transplant for multiple myeloma CALGB ECOG BMT-CTN 100104.

[19] Palumbo AP, Catalano J (2011). Incidence of second primary malignancy in melphalan-prednisone-lenalidomide combination followed by lenalidomide maintenance in newly diagnosed multiple myeloma patients age 65 or older [abstract]. J Clin Oncol.

[20] Guthrie TH (1987). High-dose, continuous-infusion cyclophosphamide, cytarabine, vincristine, and prednisone for remission induction in refractory adult acute leukemia. Cancer.

[21] Nooka AK, Kaufman JL, Behera M (2013). Bortezomib-containing induction regimens in transplant-eligible myeloma patients: a meta-analysis of phase 3 randomized clinical trials. Cancer.

[22] Richardson PG, Schlossman RL, Alsina M (2013). PANORAMA 2: panobinostat in combination with bortezomib and dexamethasone in patients with relapsed and bortezomib-refractory myeloma. Blood.

[23] Gay F, Hayman SR, Lacy MQ (2010). Lenalidomide plus dexamethasone versus thalidomide plus dexamethasone in newly diagnosed multiple myeloma: a comparative analysis of 411 patients. Blood.

[24] Dimopoulos MA, Delforge M, Hájek R (2013). Lenalidomide, melphalan, and prednisone, followed by lenalidomide maintenance, improves health-related quality of life in newly diagnosed multiple myeloma patients aged 65 years or older: results of a randomized phase III trial. Haematologica.

[25] Razavi P, Rand KA, Cozen W (2013). Patterns of second primary malignancy risk in multiple myeloma patients before and after the introduction of novel therapeutics. Blood Cancer J.

[26] Thomas A, Mailankody S, Korde N (2012). Second malignancies after multiple myeloma: from 1960s to 2010s. Blood.

[27] Greene MH, Harris EL, Gershenson DM (1986). Melphalan may be a more potent leukemogen than cyclophosphamide. Ann Intern Med.

[28] Usmani SZ, Sexton R, Hoering A (2012). Second malignancies in total therapy 2 and 3 for newly diagnosed multiple myeloma: influence of thalidomide and lenalidomide during maintenance. Blood.

